# Deregulation of Astroglial TASK-1 K^+^ Channel Decreases the Responsiveness to Perampanel-Induced AMPA Receptor Inhibition in Chronic Epilepsy Rats

**DOI:** 10.3390/ijms24065491

**Published:** 2023-03-13

**Authors:** Duk-Shin Lee, Tae-Hyun Kim, Hana Park, Tae-Cheon Kang

**Affiliations:** 1 Department of Anatomy and Neurobiology, College of Medicine, Hallym University, Chuncheon 24252, Republic of Korea; 2 Institute of Epilepsy Research, College of Medicine, Hallym University, Chuncheon 24252, Republic of Korea

**Keywords:** astrocyte, intractable epilepsy, ml365, pharmacoresistant epilepsy, refractory seizure

## Abstract

**T**andem of P domains in a weak inwardly rectifying K^+^ channel (TWIK)-related **a**cid **s**ensitive **K^+^**-1 channel (TASK-1) is activated under extracellular alkaline conditions (pH 7.2–8.2), which are upregulated in astrocytes (particularly in the CA1 region) of the hippocampi of patients with temporal lobe epilepsy and chronic epilepsy rats. Perampanel (PER) is a non-competitive α-amino-3-hydroxy-5-methylisoxazole-4-propionic acid receptor (AMPAR) antagonist used for the treatment of focal seizures and primary generalized tonic–clonic seizures. Since AMPAR activation leads to extracellular alkaline shifts, it is likely that the responsiveness to PER in the epileptic hippocampus may be relevant to astroglial TASK-1 regulation, which has been unreported. In the present study, we found that PER ameliorated astroglial TASK-1 upregulation in responders (whose seizure activities were responsive to PER), but not non-responders (whose seizure activities were not responsive to PER), in chronic epilepsy rats. ML365 (a selective TASK-1 inhibitor) diminished astroglial TASK-1 expression and seizure duration in non-responders to PER. ML365 co-treatment with PER decreased spontaneous seizure activities in non-responders to PER. These findings suggest that deregulation of astroglial TASK-1 upregulation may participate in the responsiveness to PER, and that this may be a potential target to improve the efficacies of PER.

## 1. Introduction

Epilepsy is clinically characterized by the periodic and unpredictable occurrence of seizures, which are initiated by the synchronous and rhythmic firing of populations of neurons in the brain. Multifactorial events are involved in seizure generation, such as an aberrant inflammatory response, deregulations of voltage-gated ion channel and ion/neurotransmitter transporters and abnormal neuronal circuits. In particular, the imbalance between γ-aminobutyric acid (GABA)-ergic inhibition and glutamatergic excitatory transmissions has received focus as a potential factor for ictogenesis (seizure generation) [1,2,3,4]. Therefore, prevention or inhibition of neuronal hyperactivity by anti-epileptic drugs (AEDs) is the primary therapeutic strategy for epilepsy medications. Unfortunately, partial or total inefficacy of AED treatment is seen in ~30% of patients with temporal lobe epilepsy (TLE) [5,6]. The diminished yields of AED concentration in the brain by hyperactivation of drug efflux transporters, a sustained inflammatory condition and dysfunctions of ion/neurotransmitter channels of transporters are relevant to pharmacoresistant epilepsy [1,7]. However, the underlying mechanisms of AED non-responsiveness are not fully understood.

Since glutamatergic hyperexcitation causes the pathogenesis of epilepsy and the seizure-induced secondary neuronal damage, the abrogation of presynaptic glutamate release and/or postsynaptic glutamate receptor functions are therapeutic targets to inhibit ictogenesis. Perampanel (PER, 2-(2-oxo-1-phenyl-5-pyridin-2-yl-1,2-dihydropyridin-3-yl)benzonitrile) is an AED acting as a non-competitive α-amino-3-hydroxy-5-methylisoxazole-4-propionic acid receptor (AMPAR) antagonist [8]. However, PER cannot cease or decrease seizure activity in ~15–50% of TLE patients [9,10,11]. Similarly, ~30–46% of LiCl–pilocarpine chronic epilepsy rat model are not responsive to PER due to deregulation of various signaling molecules [12,13,14,15,16]. Therefore, it is important to elucidate the underlying mechanisms of refractory seizures to PER to understand the pathogenesis of intractable epilepsy as well as AMPAR-mediated neuronal excitability.

On the other hand, the change in the extracellular pH (pHo) affects a wide range of neuronal excitability by modulating receptors and channels. Although pHo ranges around ~7.35 under physiological condition, glutamate receptor activation induces alkaline shifts in the CA1 region by 0.01–0.12 pH units [17,18,19,20,21,22,23]. Extracellular alkalinization also decreases GABA type A (GABA_A_) receptor-mediated inhibition [24], but enhances N-methyl-D-aspartate receptor (NMDAR)-mediated excitatory current [25,26]. In contrast, an acidic pHo (6.5) significantly inhibits AMPAR-mediated responses in neurons [27]. Therefore, extracellular alkaline shifts initiate and/or aggravate seizure activity, while extracellular acidifications terminate and/or attenuate epileptiform discharges [28,29]. Furthermore, alkalinization induced by AMPAR activation increases extracellular K^+^ concentration ([K^+^]o), which leads to hyperexcitability of neurons by inhibiting K^+^ efflux from neurons during repolarization [21,30,31]. Since astrocytes play an important role in the redistribution of extracellular K^+^ (K^+^ buffering) [30,31], it is likely that extracellular alkalinization shifts would also influence the K^+^ buffering capacity of astrocytes.

**Figure 1:**
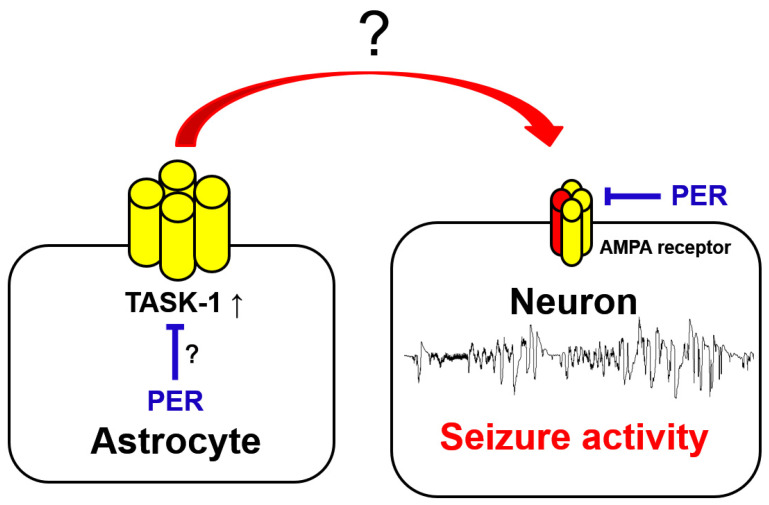
Schematic depiction representing the proposed mechanisms. The present study was conducted to investigate whether PER affects astroglial TASK-1 expression and whether aberrant TASK-1 upregulation diminishes the yield of PER in epilepsy rats. Abbreviation: AMPA receptor, α-amino-3-hydroxy-5-methylisoxazole-4-propionic acid receptor; PER, perampanel; TASK-1, Tandem of P domains in a weak inwardly rectifying K^+^ channel (TWIK)-related acid sensitive K^+^-1 channel (Red arrow and letters indicate the harmful effects of astroglial TASK-1 upregulation on epileptic seizures. Blue bars or letters indicate the possible inhibitory effects of PER in the epileptic hippocampus. ? indicate the unknown underlying mechanisms of uncontrolled seizures by PER that are investigated in the present study).

**T**andem of P domains in a weak inwardly rectifying K^+^ channel (TWIK)-related **a**cid **s**ensitive **K^+^** (TASK)-1 channel (also known as K_2P_3.1, KCNK3) is an outwardly rectifying K^+^ channel that is regulated by extracellular pH: pHo 6.0–6.4 completely inhibits TASK-1 currents, whereas pHo 7.2–8.2 potentiates them [32,33]. TASK-1 mediates neuronal depolarization and [K^+^]o under pathophysiological conditions [34,35]. Indeed, TASK-1 is upregulated in astrocytes (particularly in the CA1 region) of the hippocampi of TLE patients and chronic epilepsy rats [36,37,38]. Furthermore, the ratio of inward-to-outward K^+^ conductance in astrocytes is significantly lower in the hippocampus of TLE patients [39], and AEDs reduce astroglial TASK-1 expression in epilepsy animal models [38,40]. In particular, co-treatment with ML365 (a selective TASK-1 inhibitor) improves the responsiveness to levetiracetam (LEV, an AED) in chronic epilepsy rats [38]. Considering these previous studies, it is likely that dysregulation of astroglial TASK-1 expression in the hippocampus may be one of the causes of the inefficacy of AEDs in intractable epilepsy. To explore the underlying mechanisms of generation of refractory seizures, therefore, we investigated whether PER regulates astroglial TASK-1 upregulation and whether TASK-1 inhibition improves the efficacy of PER in epilepsy rats by applying electrophysiological, immunofluorescence and Western blot techniques (Figure 1).

Here, we demonstrate that PER reduced the increased TASK-1 expression in CA1 astrocytes of responders (whose seizure activities were responsive to PER), but not non-responders (whose seizure activities were not responsive to PER). In addition, ML365 co-treatment with PER diminished seizure activity in non-responders, concomitant with the TASK-1 downregulation. To the best of our knowledge, our findings suggest, for the first time, that dysregulation of AMPAR-TASK-1 interactions may be relevant to refractory seizures in response to PER, and TASK-1 inhibition may improve the responsiveness to PER.

## 2. Results

**Figure 2:**
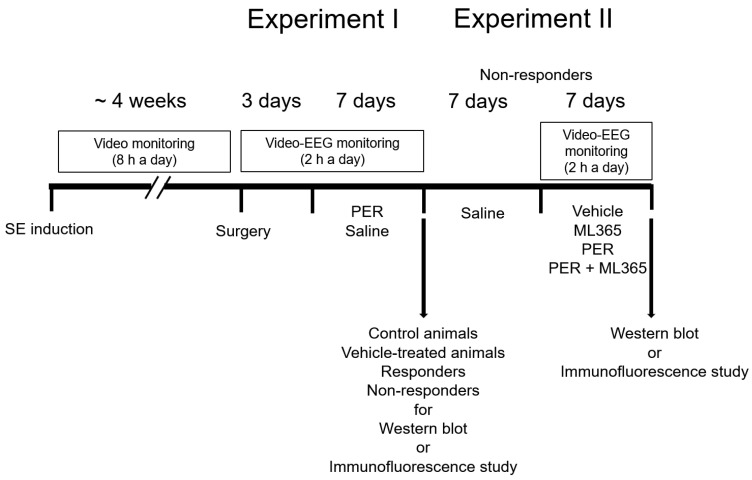
Scheme of the experimental design in the present study. After responders and non-responders were selected, they were used for Western blot or immunofluorescence studies together with control (normal) and vehicle-treated epilepsy rats. Some non-responders were intracerebroventricularly infused with vehicle or ML365. Some animals in this group were also daily given vehicle or PER. After recording, animals were used for Western blot or immunofluorescence studies. Abbreviation: EEG, electroencephalogram; ML365, a specific TASK-1 inhibitor; PER, perampanel; SE, status epilepticus.

Figure 2 illustrates the drug trial design in the present study based on our previous studies [14,15,16,38,41]. Briefly, PER or saline (vehicle) was administered daily to select responders and non-responders. Thereafter, animals were used for Western blot or immunofluorescence studies. Some non-responders were given saline over a 7-day period. Thereafter, rats were intracerebroventricularly infused with vehicle or ML365. Some animals in this group were also daily given vehicle or PER. After recording (18 h after the last drug treatment), animals were used for Western blot or immunofluorescence studies.

### 2.1. Efficacy of PER on Seizure Activity in Chronic Epilepsy Rats

**Figure 3:**
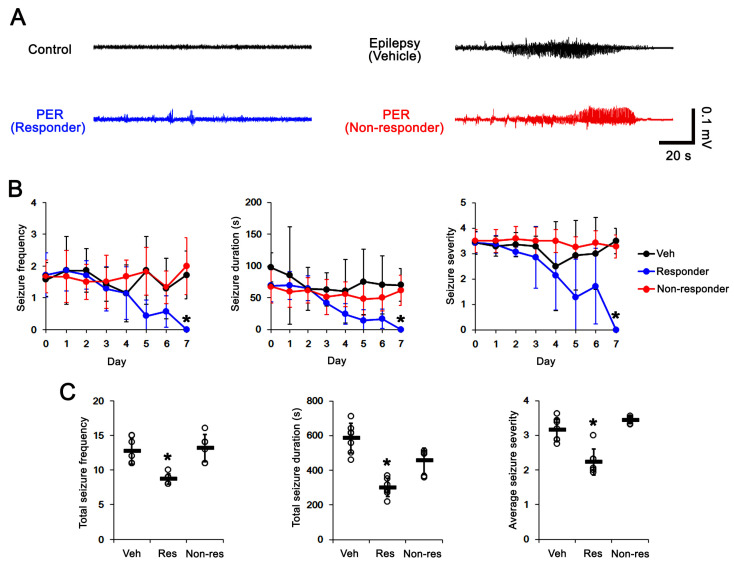
The effects of perampanel (PER) on spontaneous seizure activities in chronic epilepsy rats. PER effectively attenuates spontaneous seizure activities in responders. (**A**) Representative electroencephalograms (EEG) in each group at 4 days after treatment. (**B**) Quantitative analyses of the chronological effects of PER on seizure frequency, seizure duration and seizure severity (seizure score) over the 7-day period. Error bars indicate SD (* *p* < 0.05 vs. vehicle (Veh)-treated animals). (**C**) Quantitative analyses of total seizure frequency, total seizure duration and average behavioral seizure score (seizure severity) over the 7-day period. Open circles indicate each individual value. Horizontal bars indicate the mean value. Error bars indicate SD (* *p* < 0.05 vs. vehicle-treated animals).

First, we explored the effect of PER on spontaneous seizure activities in chronic epilepsy rats to identify responders and non-responders. In vehicle-treated epileptic rats (*n* = 7), total seizure frequency (number of seizures), total electroencephalographic (EEG) seizure duration and average seizure severity (behavioral seizure core) were 12.7 ± 1.9, 586 ± 86 s and 3.2 ± 0.3 over the 1-week period, respectively (Figure 3A–C). Responders to PER (*n* = 7) showed a gradual decreases in seizure frequency (*χ*^2^_(7)_ = 27, *p* < 0.001, Friedman test), seizure duration (*F*_(7,42)_ = 13.473, *p* < 0.001, repeated measures ANOVA) and seizure severity (*χ*^2^_(7)_ = 30, *p* < 0.001, Friedman test) over the 1-week period (Figure 3A,B). PER reduced total seizure frequency, total duration and average seizure severity to 8.7 ± 0.8 (*χ*^2^_(2)_ = 13.4, *p* = 0.001, Kruskal–Wallis test with Tukey post hoc test), 300 ± 52 s (*F*_(2,17)_ = 28.2, *p* < 0.001, one-way ANOVA with Bonferroni’s post hoc test) and 2.2 ± 0.4 (*χ*^2^_(2)_ = 12.6, *p* = 0.002, Kruskal–Wallis test with Tukey post hoc test) over the 1-week period, respectively (Figure 3C). Six out of thirteen rats (46%) in the PER-treated group were identified as non-responders whose seizure frequency was uncontrolled by PER (total seizure frequency, 13.2 ± 1.9; total seizure duration, 455 ± 72 s; average seizure severity, 3.4 ± 0.1; Figure 3A–C).

### 2.2. Effects of PER on TASK-1 Expression in the Epileptic Hippocampus

**Figure 4:**
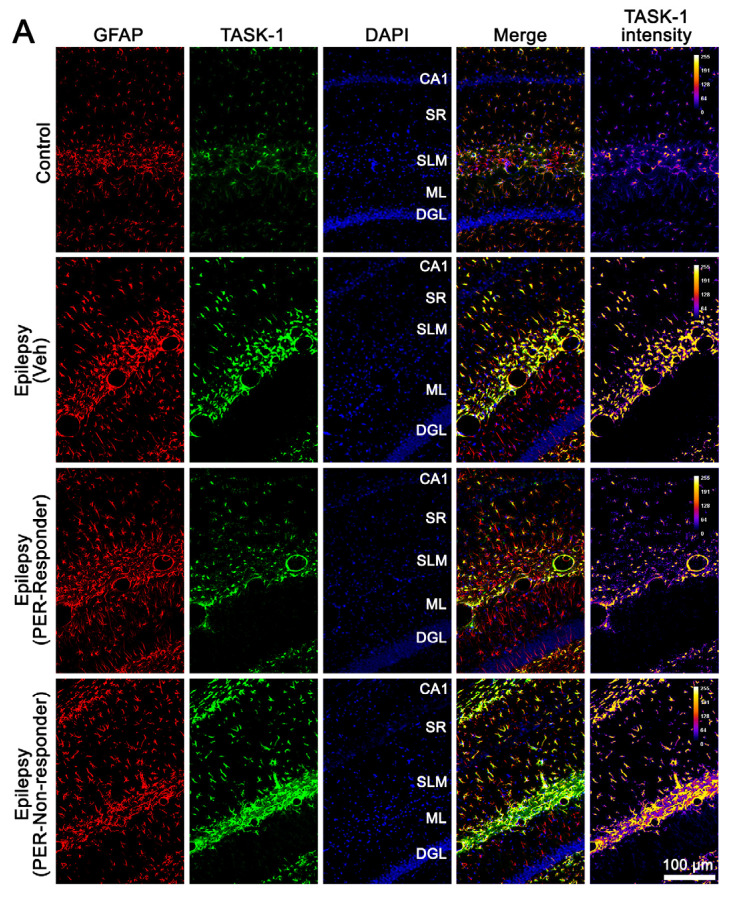

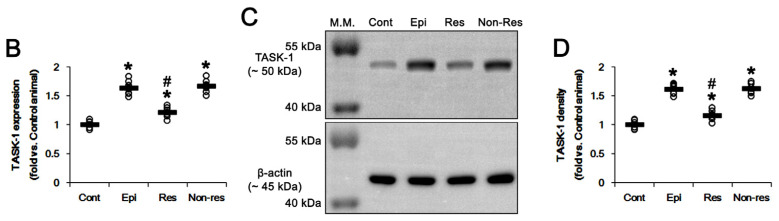
The effect of perampanel (PER) on TASK-1 expression in chronic epilepsy rats. Compared to control animals, TASK-1 expression is upregulated in CA1 astrocytes of epilepsy rats. PER significantly attenuates TASK-1 upregulation in responders, but not non-responders. (**A**) Representative photos of TASK-1 expression in the hippocampus (CA1, CA1 pyramidal cell layer; SR, stratum radiatum; SLM, stratum lacunosum-moleculare; ML, molecular layer of the dentate gyrus; DGL, dentate granule cell layer). (**B**) Quantitative analyses of the effect of PER on TASK-1 expression based on immunofluorescence data. Open circles indicate each individual value. Horizontal bars indicate the mean value. Error bars indicate SEM (*,# *p* < 0.05 vs. control and vehicle (Veh)-treated animals, respectively). (**C**) Representative images for Western blot of TASK-1 protein expression in the hippocampal tissues. (**D**) Quantifications of TASK-1 level based on Western blot (*,# *p* < 0.05 vs. control and vehicle (Veh)-treated animals).

Next, we explored whether the efficacy of PER in spontaneous seizure activity is relevant to TASK-1 expression. Consistent with previous studies [36,37,42], TASK-1 expression was prominently detected in glial fibrillary acidic protein (GFAP)-positive astrocytes within the stratum radiatum and stratum lacunosum-moleculare of the CA1 region of control rats (*n* = 7). TASK-1 expression was also detected in astrocytes in the molecular layer and the hilus of the dentate gyrus of control rats (Figure 4A). In chronic epilepsy rats (*n* = 7), TASK-1 expression was clearly detected in most reactive CA1 astrocytes (hypertrophy and hyperplasia of cell bodies and processes of astrocytes). However, TASK-1 expression was rarely observed in reactive astrocytes within the dentate gyrus (Figure 4A). TASK-1 fluorescence intensity was increased to 1.63-fold of control level (*p* < 0.001, one-way ANOVA with Bonferroni’s post hoc test; Figure 4A,B). In responders (*n* = 7), PER reduced TASK-1 fluorescence intensity to 1.21-fold of control level (*F*_(2,17)_ = 32.7, *p* < 0.001, one-way ANOVA with Bonferroni’s post hoc test), but not non-responders (*n* = 6; Figure 4A,B). Western blot also revealed that TASK-1 density was elevated to 1.62-fold of control level in chronic epilepsy rats (*p* < 0.001, one-way ANOVA with Bonferroni’s post hoc test; Figure 4C,D). PER reduced TASK-1 density to 1.16-fold of control level in responders (*F*_(2,17)_ = 55.1, *p* < 0.001, one-way ANOVA with Bonferroni’s post hoc test), but not non-responders (*n* = 6; Figure 4C,D and Supplementary Figure S1). Together with the effects of PER on seizure activity, these findings indicate that the upregulated TASK-1 expression in CA1 astrocytes may be relevant to spontaneous seizure activity and affect the responsiveness to PER.

### 2.3. Effects of ML365 on Spontaneous Seizures and TASK-1 Expression in Chronic Epilepsy Rats

**Figure 5:**
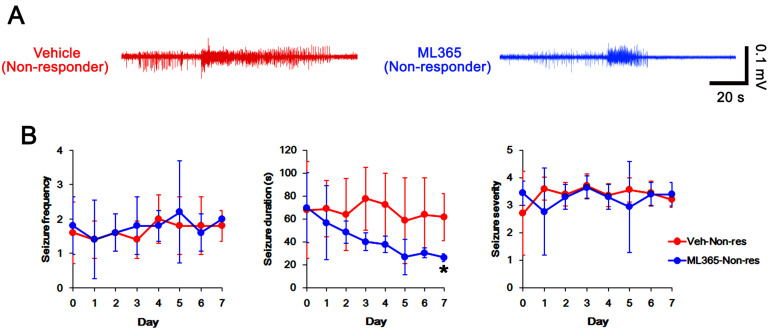

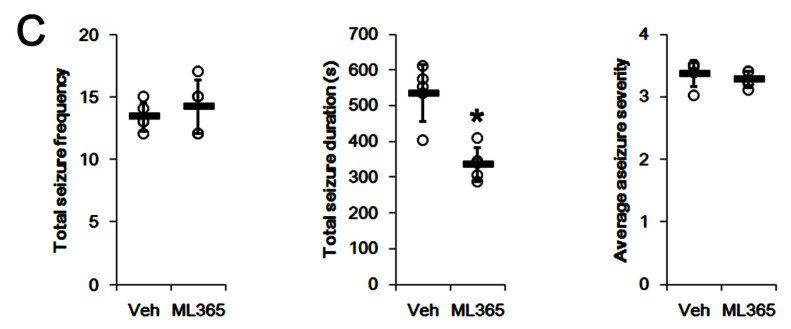
The effects of ML365 on spontaneous seizure activities in non-responders to perampanel (PER). ML365 decreases seizure duration, but not its frequency and severity. (**A**) Representative electroencephalograms (EEG) in each group at 4 days after treatment. (**B**) Quantitative analyses of the chronological effects of PER on seizure frequency, seizure duration and seizure severity (seizure score) over the 7-day period. Error bars indicate SD (* *p* < 0.05 vs. vehicle (Veh)-treated animals). (**C**) Quantitative analyses of total seizure frequency, total seizure duration and average behavioral seizure score (seizure severity) in the 7-day period. Open circles indicate each individual value. Horizontal bars indicate the mean value. Error bars indicate SD (* *p* < 0.05 vs. vehicle (Veh)-treated animals).

**Figure 6:**
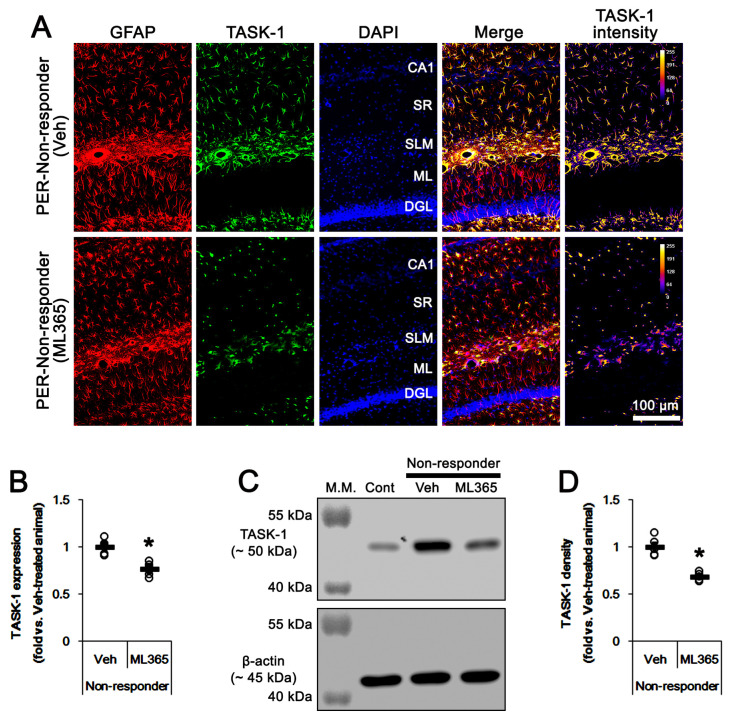
The effect of ML365 on TASK-1 expression in non-responders to perampanel (PER). As compared to vehicle (Veh), ML365 reduces TASK-1 expression in CA1 astrocytes within the hippocampus of non-responders. (**A**) Representative photos of TASK-1 expression in the hippocampus (CA1, CA1 pyramidal cell layer; SR, stratum radiatum; SLM, stratum lacunosum-moleculare; ML, molecular layer of the dentate gyrus; DGL, dentate granule cell layer). (**B**) Quantitative analyses of the effect of ML365 on TASK-1 expression based on immunofluorescence data. Open circles indicate each individual value. Horizontal bars indicate the mean value. Error bars indicate SEM (* *p* < 0.05 vs. vehicle-treated animals). (**C**) Representative Western blot images of TASK-1 protein expression in the hippocampal tissues. (**D**) Quantifications of TASK-1 level based on Western blot (* *p* < 0.05 vs. vehicle-treated animals).

To investigate the role of TASK-1 in intractable seizure activity, we examined whether TASK-1 inhibition by ML365 (a selective TASK-1 inhibitor [43]) affects spontaneous seizures in non-responders to PER. In vehicle-treated non-responders (*n* = 5), total seizure frequency, total EEG seizure duration and average seizure severity were 13.4 ± 1.1, 535 ± 79 s and 3.4 ± 0.2 over the 1-week period, respectively (Figure 5A–C). ML365 gradually decreased seizure duration (*F*_(7,28)_ = 3.8, *p* = 0.005, repeated measures ANOVA, *n* = 5), but not seizure frequency and seizure severity over the 1-week period (Figure 5A,B). Thus, ML365 diminished only total seizure duration to 336 ± 47 s over the 1-week period (*t*_(8)_ = 4.9, *p* = 0.001, Student’s *t*-test; Figure 5C). Furthermore, ML365 reduced TASK-1 fluorescence intensity and its density to 0.76- (*t*_(8)_ = 4.7, *p* = 0.002, *n* = 5, respectively, Student’s *t*-test) and 0.69-fold of vehicle levels (*t*_(8)_ = 6.5, *p* < 0.001, *n* = 5, respectively, Student’s *t*-test; Figure 6A–D and Supplementary Figure S1). These findings suggest that TASK-1 may not be involved in ictogenesis, but in sustained seizure activity in chronic epilepsy rats.

### 2.4. Effect of ML365 Co-Treatment on Refractory Seizures in Non-Responders to PER

**Figure 7:**
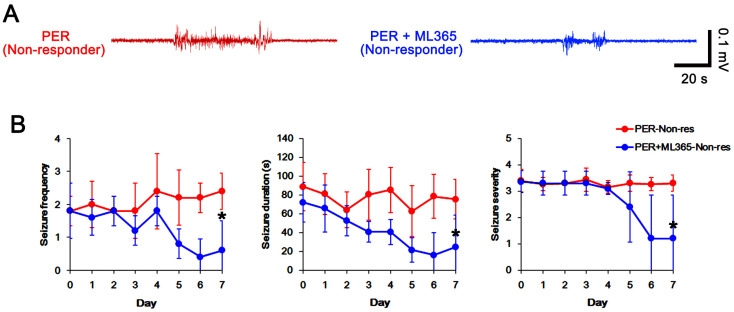

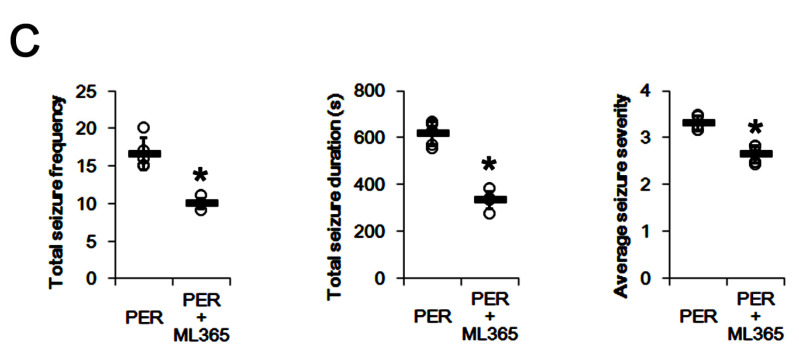
The effect of ML365 co-treatment with perampanel (PER) on spontaneous seizure activities in non-responders to PER. ML365 co-treatment improves the efficacy of PER in non-responders (**A**) Representative electroencephalograms (EEG) in each group at 4 days after treatment. (**B**) Quantitative analyses of the chronological effects of PER on seizure frequency, seizure duration and seizure severity (seizure score) over the 7-day period. Error bars indicate SD (* *p* < 0.05 vs. PER-treated animals). (**C**) Quantitative analyses of total seizure frequency, total seizure duration and average behavioral seizure score (seizure severity) in the 7-day period. Open circles indicate each individual value. Horizontal bars indicate the mean value. Error bars indicate SD (* *p* < 0.05 vs. PER-treated animals).

**Figure 8:**
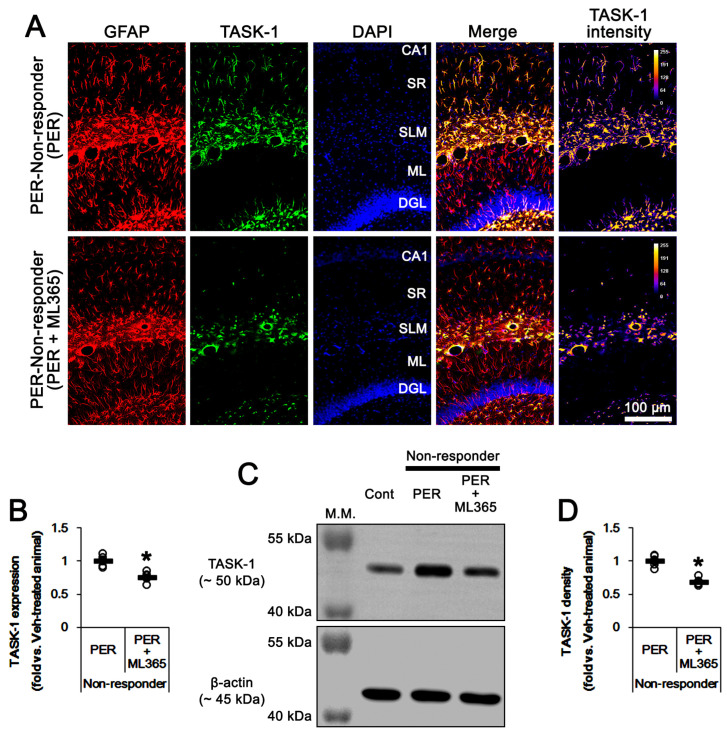
The effect of ML365 co-treatment with perampanel (PER) on TASK-1 expression in non-responders to PER. ML365 co-treatment reduces TASK-1 expression in CA1 astrocytes within the hippocampus of non-responders. (**A**) Representative photos of TASK-1 expression in the hippocampus (CA1, CA1 pyramidal cell layer; SR, stratum radiatum; SLM, stratum lacunosum-moleculare; ML, molecular layer of the dentate gyrus; DGL, dentate granule cell layer). (**B**) Quantitative analyses of the effect of ML365 co-treatment on TASK-1 expression based on immunofluorescence data. Open circles indicate each individual value. Horizontal bars indicate the mean value. Error bars indicate SEM (* *p* < 0.05 vs. perampanel-treated animals). (**C**) Representative Western blot images of TASK-1 protein expression in the hippocampal tissues. (**D**) Quantifications of TASK-1 level based on Western blot (* *p* < 0.05 vs. perampanel-treated animals).

Since PER and ML365 effectively reduced TASK-1 expression in CA1 astrocytes (Figure 4 and Figure 6), it is likely that upregulated TASK-1 function/expression in CA1 astrocytes would reduce the efficacy of PER in chronic epilepsy rats. Therefore, we evaluated the effects of ML365 co-treatment on intractable seizures in non-responders to PER. In non-responders to PER (*n* = 5), total seizure frequency, total EEG seizure duration and average seizure severity were 16.6 ± 2.1, 616 ± 51 s and 3.3 ± 0.2 over the 1-week period, respectively (Figure 7A–C). ML365 co-treatment gradually reduced seizure frequency (*χ*^2^_(7)_ = 17.5, *p* = 0.014, *n* = 5, Friedman test), seizure duration (*F*_(7,28)_ = 4.5, *p* = 0.002, *n* = 5, repeated measures ANOVA) and seizure severity (*χ*^2^_(7)_ = 16, *p* = 0.025, *n* = 5, Friedman test) in non-responders over the 1-week period (Figure 7A,B). Although ML365 co-treatment did not completely inhibit spontaneous seizure activity (Figure 7A,B), ML365-co-treatment diminished total seizure frequency to 10 ± 0.7 (Z = 2.7, *p* = 0.008, Mann–Whitney U test), total seizure duration to 334 ± 38 s (*t*_(8)_ = 9.9, *p* < 0.001, Student’s *t*-test), and average seizure severity to 2.7 ± 0.2 (Z = 2.6, *p* = 0.009, Mann–Whitney U test) over the 1-week period (Figure 7C). In addition, ML365 co-treatment decreased TASK-1 fluorescence intensity and its density to 0.76- (*t*_(8)_ = 4.5, *p* = 0.002, *n* = 5, respectively, Student’s *t*-test) and 0.68-fold of PER levels (*t*_(8)_ = 6.6, *p* < 0.001, *n* = 5, respectively, Student’s *t*-test) in non-responders (Figure 8A–D and Supplementary Figure S1). Taken together, our findings suggest that that dysregulated TASK-1 function/expression in CA1 astrocytes may reduce the responsiveness to PER, and that TASK-1 inhibition by ML365 may improve the efficacy of PER in non-responders. Table 1 presents a summary of the values of each treatment in the present study.

## 3. Discussion

The aim of the present study was to gain insights into the role of TASK-1 in intractable seizures to PER. The novel findings in the present study are that PER reduced TASK-1 upregulation in CA1 astrocytes of responders, but not non-responders. In addition, ML365 co-treatment effectively decreased seizure activity in non-responders, concomitant with the TASK-1 downregulation. Therefore, our findings suggest that dysregulation of TASK-1 expression in CA1 astrocytes may be relevant to the reduced efficacy of PER in epileptic seizures.

The AMPAR is one of the major therapeutic targets for TLE medication [44]. AMPAR antagonists, but not NMDAR antagonists, terminate status epilepticus (SE, a prolonged seizure activity) and seizure activity in animal models, and preferentially suppress seizure-related long-term consequences [45,46,47]. However, ~15–50% of patients with epilepsy are non-responders to PER [9,10,11]. Similarly, ~30–46% of chronic epilepsy rats are non-responders to PER [12,13,14,15,16]. Compatible with these previous reports, the present data also show that 46% of chronic epilepsy rats were non-responders to PER. Intensive neuronal activity, such as seizures, induces extracellular alkalinization and a subsequent acidic shift in the CA1 region [28,29,48,49]. Extracellular alkalinization inhibits GABA_A_ receptor-mediated inhibitory transmission and increases NMDAR-mediated current [24,25,26]. Therefore, it is generally accepted that activity-dependent pHo shifts may play an important role in ictogenesis. AMPAR activation also results in alkaline transients by ~0.07 pH units [20], while an acidic pH (6.5) significantly inhibits AMPAR-mediated currents [27]. Furthermore, the synaptic-evoked extracellular alkaline shifts are inhibited by AMPAR antagonists [19,50]. Although in the present study we could not directly measure the effects of PER on pHo in the hippocampi of responders and non-responders, it is likely that the lower responsiveness to PER in non-responders may be relevant to the impaired extracellular pH acidification. This is because low extracellular pHo-induced TASK-1 inhibition increases electrochemical driving forces of inward Na^+^ currents rather than passive outward K^+^ currents [32,33,51]. With respect to these previous reports, the present data indicate that the prolonged AMPAR activation by lower efficacies of PER in non-responders may lead to sustained extracellular alkalinization rather than acidification, which would potentiate TASK-1-mediated K^+^ efflux from astrocytes and reduce inward Na^+^ currents into astrocytes, and subsequently evoke epileptiform discharges by the increased [K^+^]o and [Na^+^]o. Indeed, alkalinization induced by AMPAR activation increases [K^+^]o [21], which leads to hyperexcitability of neurons by inhibiting K^+^ efflux from neurons during repolarization [30,31]. Furthermore, the present data show that ML365 co-treatment increased the responsiveness to PER in non-responders. Therefore, our findings suggest that TASK-1 may play an important role in the responsiveness to PER by regulating [K^+^]o.

Interestingly, the present study reveals that ML365 alone decreased seizure duration, but not seizure frequency and seizure severity, in non-responders. Although the direct experimental evidence for a role of TASK-1 in seizure duration is currently limited, it is noteworthy that astrocytes increase their internal [K^+^] and release it in a [K^+^]o-dependent manner [52], and that TASK-1 is outwardly rectifying K^+^ channels to regulate background [K^+^]o and resting membrane potential in response to pHo alkalinization [32,33]. Therefore, it is likely that TASK-1 inhibition by ML365 may abolish K^+^ efflux from astrocytes leading to the subsequent [K^+^]o reduction, which decreases the duration and propagation of synchronous discharges in the epileptic hippocampus. Indeed, the delayed clearance of K^+^ from the extracellular space by astrocytes increases seizure duration [53]. Therefore, our findings suggest that TASK-1 inhibition may improve the responsiveness to PER in the epileptic hippocampus by abrogating the prolongation of seizure activity rather than ictogenesis itself.

On the other hand, conventional AEDs (gabapentin, lamotrigine, topiramate, valproic acid, carbamazepine, vigabatrin and LEV) reduce astroglial TASK-1 expression in the epileptic hippocampus [38,40]. The present data also demonstrate that PER effectively diminished astroglial TASK-1 expression in responders. Furthermore, ML365 alone and ML365 co-treatment with PER decreased TASK-1 upregulation in non-responders. These findings indicate that the regulation of astroglial TASK-1 expression may also play an important role in the responsiveness to PER. Recently, we have reported that serum- and glucocorticoid-inducible kinase (SGK) activity (phosphorylation) is lower in the epileptic hippocampus that that in normal one. In addition, AMPAR antagonists including PER increase SGK activity via protein phosphatase 2B (PP2B)-extracellular signal-regulated kinase 1/2 (ERK1/2)-mediated signaling pathway in the hippocampus of responders [16]. Since SGK activation reduces TASK-1 current and its surface expression [54], it is plausible that PER may downregulate TASK-1 expression via SGK-mediated signaling pathway in astrocytes, although the mechanisms underlying the regulation of TASK-1 expression are largely unknown. On the other hand, dexamethasone (a potential anti-inflammatory steroid hormone) increases SGK expression and its phosphorylation [55,56]. Considering the anti-inflammatory properties of PER and ML365 [57,58,59], PER and ML365 may also indirectly activate SGK-mediated TASK-1 downregulation by regulating inflammatory responses in the epileptic hippocampus. An important avenue for future investigations is to determine the underlying mechanisms of the regulation of TASK-1 expression.

The limitations of the present study are the small sample size of the animal numbers in each group and the large error bars (SD) in the effect of ML365 co-treatment on refractory seizures in non-responders to PER. Since SD represents the variability of the observation, the large error bars may be due to the small sample size in each group. Furthermore, we cannot exclude that the broad spectrum of the responsiveness of ML365 in non-responders would also lead to these results. Further studies are needed to overcome these limitations.

## 4. Materials and Methods

### 4.1. Experimental Animals and Chemicals

This study utilized the progeny of male Sprague Dawley (SD) rats (7 weeks old). This is because estrous cycle affects the function of the hippocampus and seizure activity [60,61,62]. The animals were housed under controlled conditions (22 ± 2 °C, 55 ± 5% and a 12:12 light/dark cycle with lights) and provided with a commercial diet and water ad libitum. Animal experimental protocols were approved by the Institutional Animal Care and Use Committee of Hallym University (No. Hallym 2018-2, 26 April 2018; No. Hallym 2018-21, 8 June 2018 and No. Hallym 2021-3, 27 April 2021). All reagents were obtained from Sigma-Aldrich (St. Louis, MO, USA), unless otherwise described.

### 4.2. Generation of Chronic Epilepsy Rats

Rats were given LiCl (127 mg/kg, i.p.) 24 h before the pilocarpine treatment. Animals were intraperitoneally (i.p.) treated with pilocarpine (30 mg/kg) 20 min prior to atropine methylbromide (5 mg/kg, i.p.) treatment. Diazepam (Hoffman la Roche, Neuilly-sur-Seine, France; 10 mg/kg, i.p.) was administered 2 h after onset of SE and repeated as needed. Control animals received saline in place of pilocarpine. The volume of each solution administered was 0.2 mL. Thereafter, rats were video-monitored 8 h a day for general behavior and occurrence of spontaneous seizures for 4 weeks after SE [14,15,38,41]. Chronic epilepsy rats were classified by behavioral seizures with seizure score ≥3 more than once, according to Racine’s scale [63].

### 4.3. Surgery

Control and epilepsy rats were implanted with monopolar electrodes (Plastics One, Roanoke, VA, USA) in the right hippocampus (stereotaxic coordinates were −3.8 mm posterior; 2.0 mm lateral; −2.6 mm depth to bregma) under isoflurane anesthesia (3% induction, 1.5–2% for surgery, and 1.5% maintenance in a 65:35 mixture of N_2_O:O_2_). Some rats were also implanted with an infusion needle (Brain Infusion Kit 1, Alzet, Cupertino, CA, USA) into the right lateral ventricle (1 mm posterior; 1.5 mm lateral; −3.5 mm depth to the bregma, see below). Throughout surgery, the core temperature of each rat was maintained at 37–38 °C. The electrode was secured to the exposed skull with dental acrylic [14,15,38,41].

### 4.4. Drug Trial Protocols

#### 4.4.1. Experiment I

After baseline seizure activity was determined over 3 days, PER (8 mg/kg, i.p., Eisai Korea Inc., Seoul, Republic of Korea) or saline (vehicle) was administered daily at 6:00 p.m. over a 1-week period [14,15,38,41]. The volume of each solution administered was 0.2 mL. EEG signals were detected with a DAM 80 differential amplifier (0.1–1000 Hz bandpass; World Precision Instruments, Sarasota, FL, USA) 2 h a day at the same time over a 1-week period. The data were digitized and analyzed using LabChart Pro v7 (ADInstruments, Bella Vista, New South Wales, Australia). Behavioral seizure severity was measured according to Racine’s scale, as previously mentioned. Non-responders were defined as showing no reduction in seizure frequency during recording, as compared with the pre-treatment stage. Normal rats (*n* = 14) were also used as controls. After recording (18 h after the last drug treatment), animals were used for Western blot (control rats, *n* = 7; vehicle-treated rats, *n* = 7; responder, *n* = 7; non-responder, *n* = 6, respectively). Other rats were used for immunofluorescence study (control rats, *n* = 7; vehicle-treated rats, *n* = 7; responder, *n* = 7; non-responder, *n* = 6, respectively).

#### 4.4.2. Experiment II

Non-responders in experiment I were given saline (i.p.) over a 7-day period. Thereafter, rats were connected to an Alzet 1007D osmotic pump (Alzet, Cupertino, CA, USA) to infuse with vehicle or ML365 (a specific TASK-1 inhibitor, 400 nM [43], vehicle-infused rats, *n* = 10; ML365-infused rats, *n* = 10, respectively). Other animals (vehicle-infused rats, *n* = 10; ML365-infused rats, *n* = 10, respectively) were also given daily vehicle or PER by the aforementioned method (*n* = 5 in each treatment, respectively). The volume of each solution administered was 0.2 mL. After recording (18 h after the last drug treatment), animals were used for Western blot or immunofluorescence studies (*n* = 5 in each group).

### 4.5. Western Blot

Animals were sacrificed by decapitation, and their hippocampi were obtained and homogenized in radioimmunoprecipitation assay (RIPA) buffer (Thermo Fisher Scientific Korea, Seoul, Republic of Korea) containing protease inhibitor cocktail (Roche Applied Sciences, Branford, CT, USA) and phosphatase inhibitor cocktail (PhosSTOP^®^, Roche Applied Science, Branford, CT, USA). Thereafter, total protein concentration was calibrated using a Micro BCA Protein Assay Kit (Pierce Chemical, Rockford, IL, USA). Western blot was performed by the standard protocol: Sample proteins (10 μg) were separated on a Bis-Tris sodium dodecyl sulfate–polyacrylamide (SDS–PAGE) gel and transferred to membranes. Membranes were incubated with 2% bovine serum albumin (BSA) in Tris-buffered saline (TBS; 10 mM Tris, 150 mM NaCl, pH 7.5, and 0.05% Tween 20), and then incubated with primary antibodies (Table 2) overnight at 4 °C. After washing, membranes were incubated in a solution containing horseradish peroxidase (HRP)-conjugated secondary antibodies for 1 h at room temperature. Immunoblots were detected and quantified using the ImageQuant LAS4000 system (GE Healthcare Korea, Seoul, Republic of Korea). Optical densities of proteins were calculated with the corresponding amount of β-actin [14,15,38,41].

### 4.6. Immunofluorescence Study

Animals were sacrificed under deep anesthesia with urethane (1.5 g/kg, i.p.). Rats were perfused with 4% paraformaldehyde via the ascending aorta. The brains were then removed, immersed in the same fixative overnight and cryoprotected in 30% sucrose in phosphate buffer (PB). Coronal sections (30 μm) of the brain samples were cut using a cryostat. After rinses with phosphate-buffered saline (PBS) over 10 min and subsequent blocking with 10% goat serum (Vector, Burlingame, CA, USA) for 30 min at room temperature, tissues were reacted with primary antibodies overnight at 4 °C (Table 2). Sections were then washed over 10 min three times with PBS and incubated with appropriate secondary antibodies for 1 h at room temperature. Brain sections incubated with pre-immune serum (for GFAP) or primary antibody reacted with control peptide (for TASK-1) were used as negative controls [36].

For quantification, five hippocampal sections from each animal were randomly captured and areas of interest (1 × 10^5^ μm^2^) were selected from the CA1 region. Thereafter, fluorescence intensity was measured using AxioVision Rel. 4.8 and ImageJ software (1.53t). The investigators were blinded to experimental groups when performing morphological analysis and immunofluorescence experiments [14,15].

### 4.7. Data Analysis

Seizure parameters (frequency, duration and Racine scores) were assessed by different investigators who were blind to the classification of animal groups and treatments. Shapiro–Wilk W test was used to evaluate the values on normality. Student’s *t*-test, Mann–Whitney U test, repeated measures ANOVA, Friedman test and Kruskal–Wallis test with Tukey post hoc comparisons and one-way ANOVA followed by Bonferroni’s post hoc comparisons. A *p*-value less than 0.05 was considered to be significant.

## 5. Conclusions

**Figure 9:**
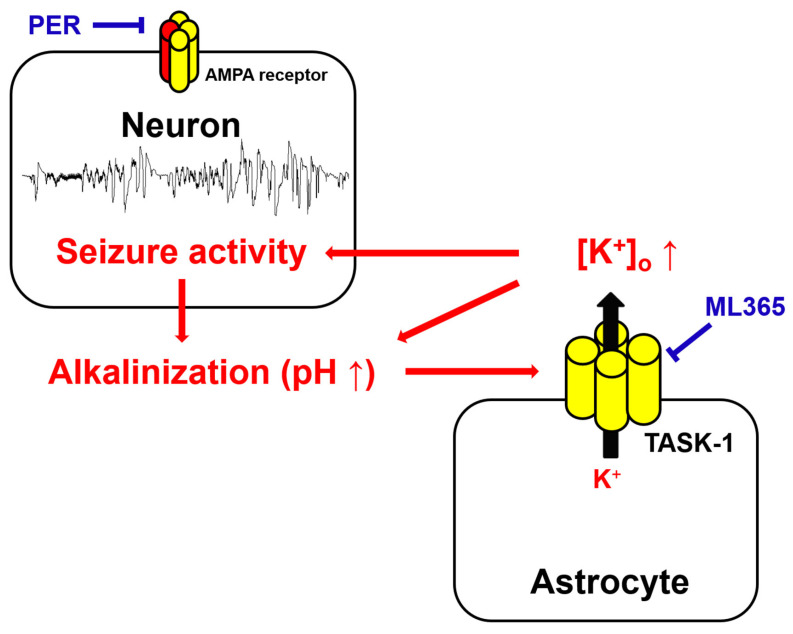
Schematic depiction representing the role of AMPAR–TASK-1 interactions in seizure activity. AMPAR hyperactivation generates seizure activity leading to extracellular alkaline shifts. Under this condition, astroglial TASK-1 channels may be activated and subsequently increase K^+^ efflux from astrocytes. In turn, TASK-1-channel-mediated extracellular K^+^ elevation further augments seizure activity (Red arrows or letters indicate the underlying mechanisms of uncontrolled seizures in response to PER. Blue bars or letters indicate the inhibitor of AMPA receptor and TASK-1 channel to cease the spontaneous seizures).

In the present study, we demonstrated, for the first time, that that TASK-1 inhibition improved the efficacy of PER in non-responders, and that the upregulated TASK-1 expression in CA1 astrocytes prolonged seizure duration, although it did not affect the generation of seizure activity. Therefore, our findings suggest that dysregulation of astroglial TASK-1 function may be involved in intractable seizures to PER, and TASK-1 inhibition may be one of the therapeutic targets for refractory TLE medications (Figure 9).

## Figures and Tables

**Table 1:** Summary of the values of each treatment in the present study (mean ± SD).

	Control Rats	Epilepsy Rats						
		Veh	Res (PER)	Non-Res (PER)	Non-Res (Veh)	Non-Res (ML365)	Non-Res (PER+Veh)	Non-Res (PER+ML365)
Total seizure freqeuncy	0	12.71 ± 1.89	8.71 ± 0.76 ^a^*	13.17 ± 1.94	13.4 ± 1.14	14.2 ± 2.17	16.6 ± 2.07	10 ± 0.71 ^c^*
Total seizure duration (s)	0	585.71 ± 85.73	300.29 ± 51.53 ^a^*	454.67 ± 72.11	534.8 ± 78.95	335.6 ± 46.7 ^b^*	616 ± 50.81	333.6 ± 38.32 ^c^*
Average seizure severity	0	3.16 ± 0.33	2.23 ± 0.37 ^a^*	3.44 ± 0.1	3.37 ± 0.21	3.27 ± 0.13	3.3 ± 0.16	2.65 ± 0.18 ^c^*
TASK-1 expression	1 ± 0.07	1.63 ± 0.12 ^a^	1.21 ± 0.09 ^a^*	1.67 ± 0.13 ^a^	1 ± 0.08	0.76 ± 0.08 ^b^*	1 ± 0.09	0.76 ± 0.08 ^c^*
TASK-1 density	1 ± 0.07	1.62 ± 0.09 ^a^	1.16 ± 0.08 ^a^*	1.62 ± 0.1 ^a^	1 ± 0.1	0.69 ± 0.04 ^b^*	1 ± 0.09	0.68 ± 0.06 ^c^*

Footnote: ^a^, ^b^ and ^c^ indicate the fold change over control rat, vehicle-infused non-responder and PER+vehicle-treated non-responder levels, respectively (^a^*^, b^*^, c^* *p* < 0.05 vs. vehicle-treated epilepsy animal, vehicle-infused non-responder and PER+vehicle-treated non-responder, respectively).

**Table 2:** Primary antibodies used in the present study.

Antigen	Host	Manufacturer (Catalog Number)	Dilution
Glia fibrillary acidic protein (GFAP)	Mouse	Millipore (MAB3402)	1:4000 (IF)
TASK-1	Rabbit	Millipore (AB5250)	1:50 (IF) 1:200 (WB)
β-actin	Mouse	Sigma (A5316)	1:5000 (WB)

IF, immunofluorescence; WB, Western blot.

## Data Availability

Not applicable.

## Supplementary Materials

The supporting information can be downloaded at https://www.mdpi.com/article/10.3390/ijms24065491/s1.
