# Supplementary material for: Deregulation of Astroglial TASK-1 K+ Channel Decreases the Responsiveness to Perampanel-Induced AMPA Receptor Inhibition in Chronic Epilepsy Rats
Source: Int J Mol Sci. 2023 Mar 13;24(6):5491. doi: 10.3390/ijms24065491 (PMC10049714; doi:10.3390/ijms24065491)
Supplement: Supplementary file 1 [file ijms-24-05491-s001.zip › ijms-2259796-supplementary.pdf]

Supplementary Information

Deregulation of astroglial TASK-1 K^+ channel decreases the responsiveness to perampanel-induced AMPA receptor inhibition in chronic epilepsy rats

Duk-Shin Lee[1,2], Tae-Hyun Kim[1,2], Hana Park[1,2] and Tae-Cheon Kang[1,2]*

[1]Department of Anatomy and Neurobiology, Institute of Epilepsy Research, College of Medicine, Hallym University, Chuncheon 24252, Republic of Korea

* Correspondence to: T. -C. Kang, Department of Anatomy and Neurobiology, College of Medicine, Hallym University, Chuncheon, Kangwon-Do 24252, Republic of Korea; Tel: +82-33-248-2524; Fax: +82-33-248-2525; E-mail: tckang@hallym.ac.kr

Fig. 4C

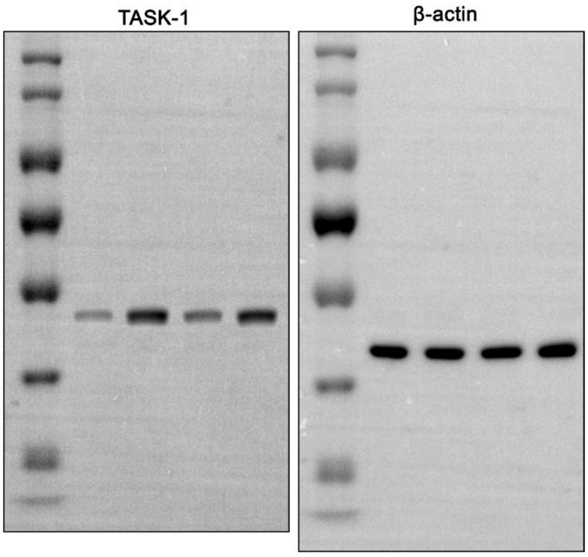

Fig. 6C

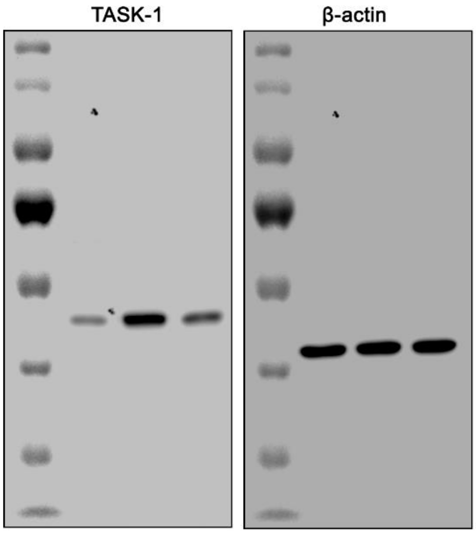

Fig. 8C

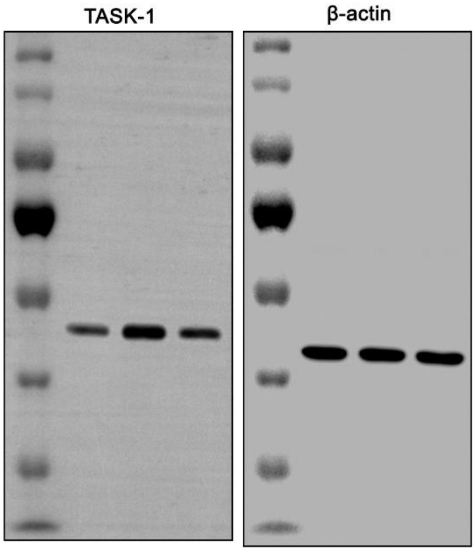

Supplementary Figure S1. Full-gel images of the Western blot data in Figs 4C, 6C and 8C.
